# Editorial: The impact of clinical and environmental toxicological exposures and eye health

**DOI:** 10.3389/ftox.2024.1344052

**Published:** 2024-02-22

**Authors:** Sneh Patel, Anat Galor

**Affiliations:** ^1^ Physical Medicine and Rehabilitation, VA Greater Los Angeles Healthcare System, Veterans Health Administration, United States Department of Veterans Affairs, Los Angeles, CA, United States; ^2^ Physical Medicine and Rehabilitation, University of California, Los Angeles, Los Angeles, CA, United States; ^3^ Ophthalmology, Miami VA Healthcare System, Veterans Health Administration, United States Department of Veterans Affairs, Miami, FL, United States; ^4^ Bascom Palmer Eye Institute, University of Miami Health System, Miami, FL, United States; ^5^ University of Miami Health System, Miami, FL, United States

**Keywords:** toxicology, environment, dry eye, medication, weather, air pollutant, temperature, humidity

Increasing focus has been placed on understanding how our environment may influence our health, with ample research being conducted towards understanding how factors such as weather, air pollution, chemicals, and medications may affect the human body. Through these efforts, we have gained a more robust understanding of how these factors may have harmful, albeit variable, effects on ocular health, depending on the exposure in question. Galor et al. (2020), Villani et al. (2020) Research has linked environmental exposures to eye disorders, such as dry eye disease (DED) (Galor et al., 2014), Sjögren’s disease (Xin et al., 2022), and allergic conjunctivitis (Patel et al., 2021), among others. In this Research Topic, we highlight environmental exposures that relate to ocular disease, with an aim of improving understanding and promoting precision-based management. A range of exposures were examined, including weather, air pollution, chemicals, and medications. Furthermore, study methodologies varied, including reviews of existing literature (de Los Santos et al.; Graca et al., 2023; Huang et al.; Ma et al.; Menke et al.; Patel et al.; Quiroga-Garza et al.; Ruiz-Lozano et al.), analyses of health outcomes (Shanbag et al.), and exposure assessment *in vivo* (Chen et al.; Tyszkiewicz et al.) and *in vitro* models. Fukuda et al..

First, Patel et al. focused on environmental exposures, such as airborne pollutants (particulate matter (PM) and reactive gases like ozone or nitrogen dioxide), meteorological conditions (temperature, relative humidity (RH)), and behavioral factors (smoking, contact lenses) and summarized how these factors relate to ocular surface disease. This article centered around risk for DED, a common source of ocular morbidity across the world. The authors concluded that these toxicological exposures related to DED risk in variable ways, depending on the exposure. DED was consistently linked to air pollution, with studies reporting positive linear relationships with various aspects of DED risk, including its diagnosis, symptoms, and clinical signs. Relationships for temperature and RH were on the other hand ‘U-shaped,’ suggesting that extremes in either direction increased the risk for these same aspects of DED.

Other authors focused on chemical exposures. Menke et al. reviewed the toxic effects of chemical agents used in threat control and civilian conflicts, like sulfur mustard, sarin, and caustic hydrofluoric acid. Ocular complications ranged from self-limiting conjunctival irritation to more severe corneal disease (neovascularization, opacification, perforation), and in some cases resulting in blindness. Graca et al. summarized the molecular and anatomic mechanisms that linked chemical injury to ocular pathology. For example, the authors described how acidic exposures are limited damage the cornea, while alkali agents can penetrate deeper layers of the eye via saponification and lysis of epithelial membranes. Furthermore, the authors explained how chemical exposures can lead to the development of chronic ocular pain even after healing at the ocular surface. This included a discussion of the ocular pain pathway, starting from nociceptive responses at the ocular surface to eventual development of central sensitization. Finally, in a review by Quiroga-Garza et al., riot control agents such as oleoresin capsicum and chloroacetophenone (components of Mace and pepper spray) were examined in relationship to the eye. While use of such agents is intended to incapacitate through transient neurogenic inflammation secondary to activation of corneal surface afferents, the authors highlighted the possibility of more severe corneal complications (e.g., necrosis and neovascularization).

A few articles also examined how non-prescription drugs may affect the eye. Huang et al. reviewed the ocular effects of methamphetamine over-use, including the risk of self-limited (e.g., conjunctivitis and keratitis) and permanent (e.g., corneal melting, amaurosis fugax, non-arteritic anterior optic ischemic neuropathy (NAION), orbital cellulitis) sequela. The authors also examined on molecular mechanisms by which methamphetamine can affect the eye, from the ocular surface to the optic nerve. In another review by de Los Santos et al., the toxic effects of mercury were examined, beyond its known deleterious effects on the central nervous system. The article highlighted the potential for damage in not only the cornea (decreased subbasal nerve density), but also within the retina (direct toxicity to photoreceptors) and optic nerve (direct toxicity to optic nerve glial cells).

Other papers examined the impact of prescription drugs on the eye. Ruiz-Lozano et al. reviewed the effects of glaucoma therapies, including outflow enhancers (e.g., prostaglandin analogs and cholinergic agents), aqueous humor blockers (e.g., beta blockers), and others (e.g., nitric-oxide modulating agents), in relation to ocular disease. The authors summarized that these medications could induce ocular pathology via lacrimal and meibomian gland dysfunction as well as direct ocular surface toxicity (contact dermatitis, drug-induced conjunctivitis). In another review by Ma et al., the impact of biologic agents used to treat cancer were examined. Epidermal growth factor receptor inhibitors and immune checkpoint inhibitors were two categories that were closely linked to eye manifestations, including ulcerative keratitis and Stevens-Johnson syndrome (SJS). A review of hydroxychloroquine-associated eye manifestations was undertaken by Yusuf et al. These authors took a step further and summarized the current diagnostic decision-making process, and postulated how we can improve this process, including with the integration of artificial intelligence, to aid in earlier diagnosis of retinopathy. Finally Shanbag et al. examined vision, pain, and quality of life in 15 individuals with chronic SJS/TENS who were treated during the acute phase of their disease using their respective hospital’s protocol (some individuals received amniotic membrane transplant). The authors examined outcomes of these 15 individuals with the National Eye Institute Visual Function Questionnaire (NEI-VFQ-25, which tests 11 subscales regarding visual acuity, social functioning, daily quality of life, ocular pain, and mental health) and found that apart from ocular pain and mental health status, all other subscale scores were comparable to those collected from 122 controls. The authors concluded that early treatment of SJS/TENS can lead to improved long-term outcomes.

This Research Topic also included experimental studies. Chen et al. utilized a mouse model to examine the impact of bisphenol A, an organic compound used in plastics and resins, on ocular health. Two groups of mice were examined—a control group, and a group that received 100 mg/kg BPA administered daily for 14 days by intraperitoneal injection. After exposure, the authors observed upregulated expression of scleral endoplasmic reticulum stress proteins associated with matrix remodeling and fibrosis (Activating transcription factor 6 [ATF6] and Protein kinase RNA (PKR)-like ER kinase [PERK]) with resultant axial lengthening and scleral remodeling in exposed eyes, suggesting that exposure may lead to an increased risk for myopia. Tyszkiewicz et al. instead examined retinal function in healthy Wistar Han rat eyes. The authors assessed male and female retinal structure and function via electroretinography (ERG, to test scotopic and photopic luminance responses of retinal cells), optokinetic responses (to assess visual acuity and tracking responses), and histology (to assess retinal, brainstem, and visual/auditory area cell structure), among others. The study found that a significant fraction of male rats (13%–19%) had abnormal ERG signals and decreased visual tracking responses, without notable changes in retinal or brain cell morphology. The authors thus cautioned that baseline sex-related differences must be considered when examining retinal function in these animals, including during toxicological exposure testing.

Finally Fukuda et al. used an *in vitro* model to study ocular toxicity. These authors examined the utility of a human corneal epithelial-derived cell line with enhanced proliferation. This cell line was developed through co-expression of a mutant cyclin-dependent-kinase 4 (CD-K4), Cyclin-D1, and telomerase reverse transcriptase (TERT) which allowed for increased proliferation. The authors challenged the cell line with glycolic acid and benzalkonium chloride, chemical preservatives often found in topical eye therapies, and noted a dose-dependent decrease in viability post-exposure. The authors concluded that this new cell line could be used to evaluate the impact of additional chemicals in future studies.

In summary, this special edition highlights that toxicological exposures, whether environmental, chemical, or drug-associated, can induce ocular disease. Pathology can range from the ocular surface to the optic nerve, and these disorders are driven by a variety of underlying mechanisms. It is important for providers to be aware of these associations as this knowledge can be incorporated into practice by discussing exposure avoidance or mitigation for susceptible patients. Rozanova et al. (2009).
